# Supplementary material for: Regulatory mechanisms of lipopolysaccharide synthesis in Escherichia coli
Source: Nat Commun. 2022 Aug 5;13:4576. doi: 10.1038/s41467-022-32277-1 (PMC9356133; doi:10.1038/s41467-022-32277-1)
Supplement: Supplementary file 1 — Supplementary Information [file 41467_2022_32277_MOESM1_ESM.pdf]

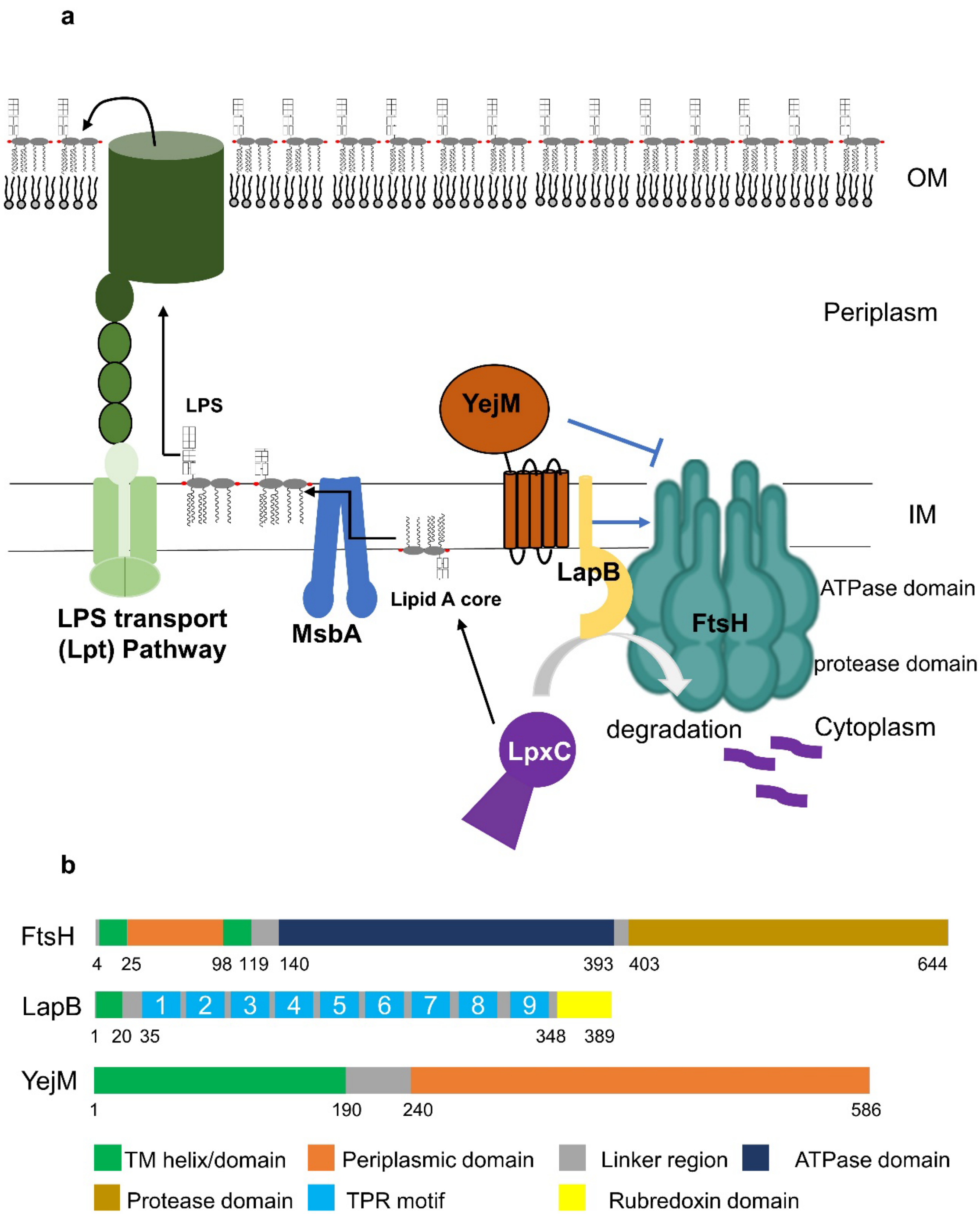

Supplementary Figure 1 | Synthesis, transport, and regulation of LPS. **a** Cartoon representation of LPS transport and regulation of its biosynthesis. Black arrows represent the pathway of LPS synthesis and transport; blue arrow represents that LapB stimulates FtsH protease activity on LpxC; blue lines represent YejM inhibits FtsH-mediated LpxC degradation; IM, inner membrane; OM, outer membrane. **b** Schematic representation of domain organization of three essential membrane proteins involved in the regulation of LPS biosynthesis: FtsH, LapB, and YejM.

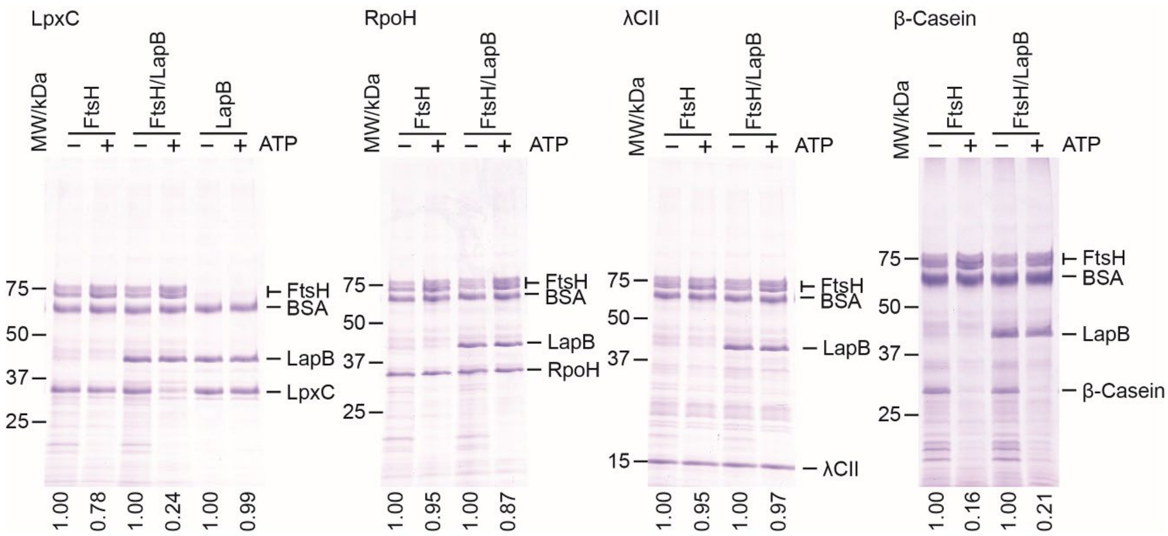

Supplementary Figure 2 | Degradation of LpxC, RpoH, CII, and β-casein by FtsH or the FtsH/LapB complex in detergent micelles. For each pair of reactions (without or with ATP), the intensities of the substrate bands are quantified under each lane, with intensities in the reaction without ATP at 1.00. Purified C-terminal His-tagged FtsH showed two bands in SDS-PAGE gels, consistent with a previous report that FtsH undergoes C-terminal self-processing without affecting its protease activity[54]. Each experiment was repeated independently three times with similar results. Source data are provided as a Source Data file.

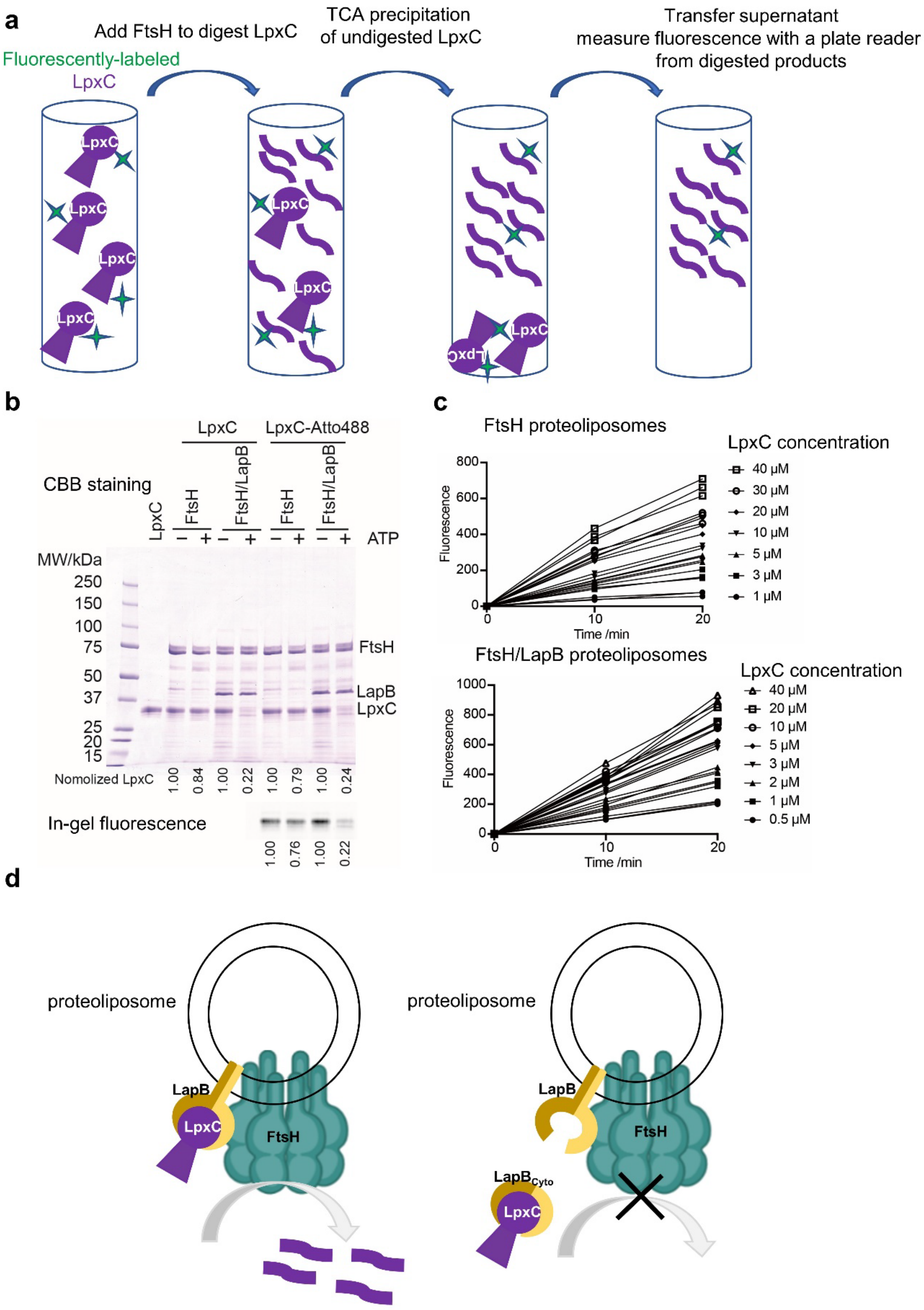

Supplementary Figure 3 | *In vitro* analysis of LapB stimulatory effect on LpxC degradation.
a Experimental design of *in vitro* LpxC degradation assay with fluorescent labeling. LpxC is

labeled with fluorescent dye Atto488 (green star), and the digestion rate is estimated by using a plate reader to measure the fluorescence signal from the digestion product of peptide fragment after undigested LpxC is precipitated by trichloroacetic acid (TCA) and separated from the peptide fragments by centrifugation. **b** SDS-PAGE analysis of the degradation of non-labeled LpxC and Atto488-labeled LpxC. The in-gel fluorescence was obtained from the same gel under fluorescence scanner before staining with coomassie brilliant blue (CBB). For each pair of reactions (without or with ATP), the densities of the bands from respective substrates are quantified under each lane, with intensities in the reaction without ATP set to 1.00. **c** Fluorescence signal from the formation of Atto488-labeled peptide segments measured by a plate reader. The initial rates of LpxC degradation under various concentrations are estimated by the increase of fluorescence signal as reactions proceed. Each experiment was repeated three times. **d** Experimental design of inhibition by $LapB_{cyto}$ in the assay with FtsH/LapB proteoliposomes. Left, without $LapB_{cyto}$ in solution, the reconstituted FtsH/LapB complex degrades LpxC. Right, $LapB_{cyto}$ in the solution binds to the substrate LpxC, and prevents LpxC from being recruited to the FtsH/LapB proteoliposomes for degradation. Source data for **c** are provided as a Source Data file.

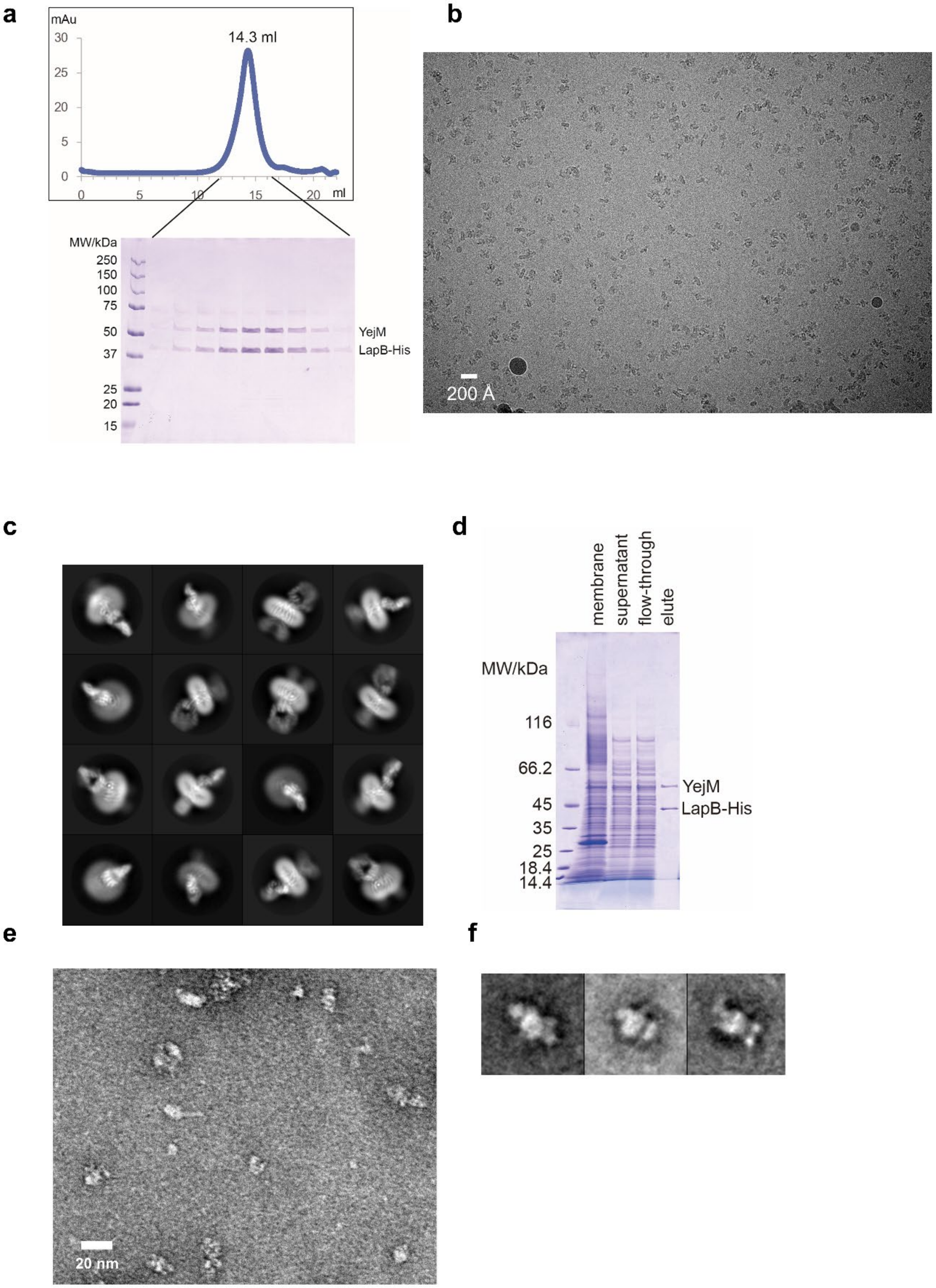

Supplementary Figure 4 | Purification of the YejM/LapB complex, EM images, and two-dimensional (2D) classification averages. a Size exclusion chromatography of the YejM/LapB

complex in detergent GDN and SDS-PAGE analysis of fractions across the peak. Each experiment was repeated independently three times with similar results. **b** A representative of cryoEM image of the purified YejM/LapB complex in GDN after motion correction from 26362 images. **c** Selected class averages from 2D classification of particles from cryoEM images. **d** SDS-PAGE analysis of YejM/LapB complex purified with SMA. Each experiment was repeated independently three times with similar results. **e** A representative of negative stain EM image of the YejM/LapB complex in SMA from more than 40 images. **f** Selected 2D class averages particles of negative stain YejM/LapB complex in SMA.

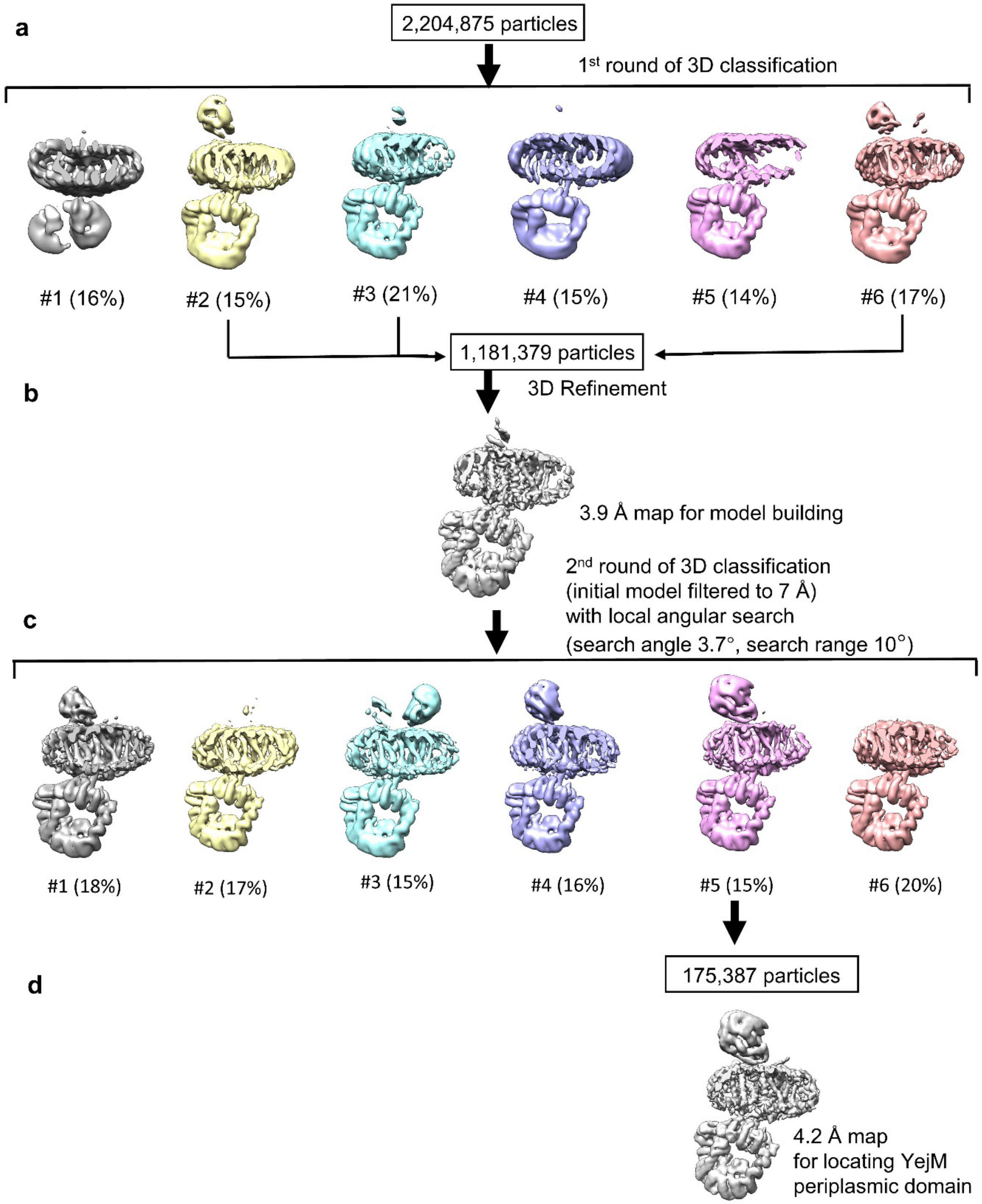

Supplementary Figure 5 | Image processing workflow for the YejM/LapB complex structure determination. **a** Initial 3D classification with RELION default parameters to remove bad particles. **b** 3D reconstruction of particles from the selected classes in the initial 3D classification with CryoSPARC non-uniform refinement. **c** A second round of 3D classification with local angular search by RELION. **d** 3D reconstruction of selected particles from class #5 in the second-round classification with CryoSPARC non-uniform refinement.

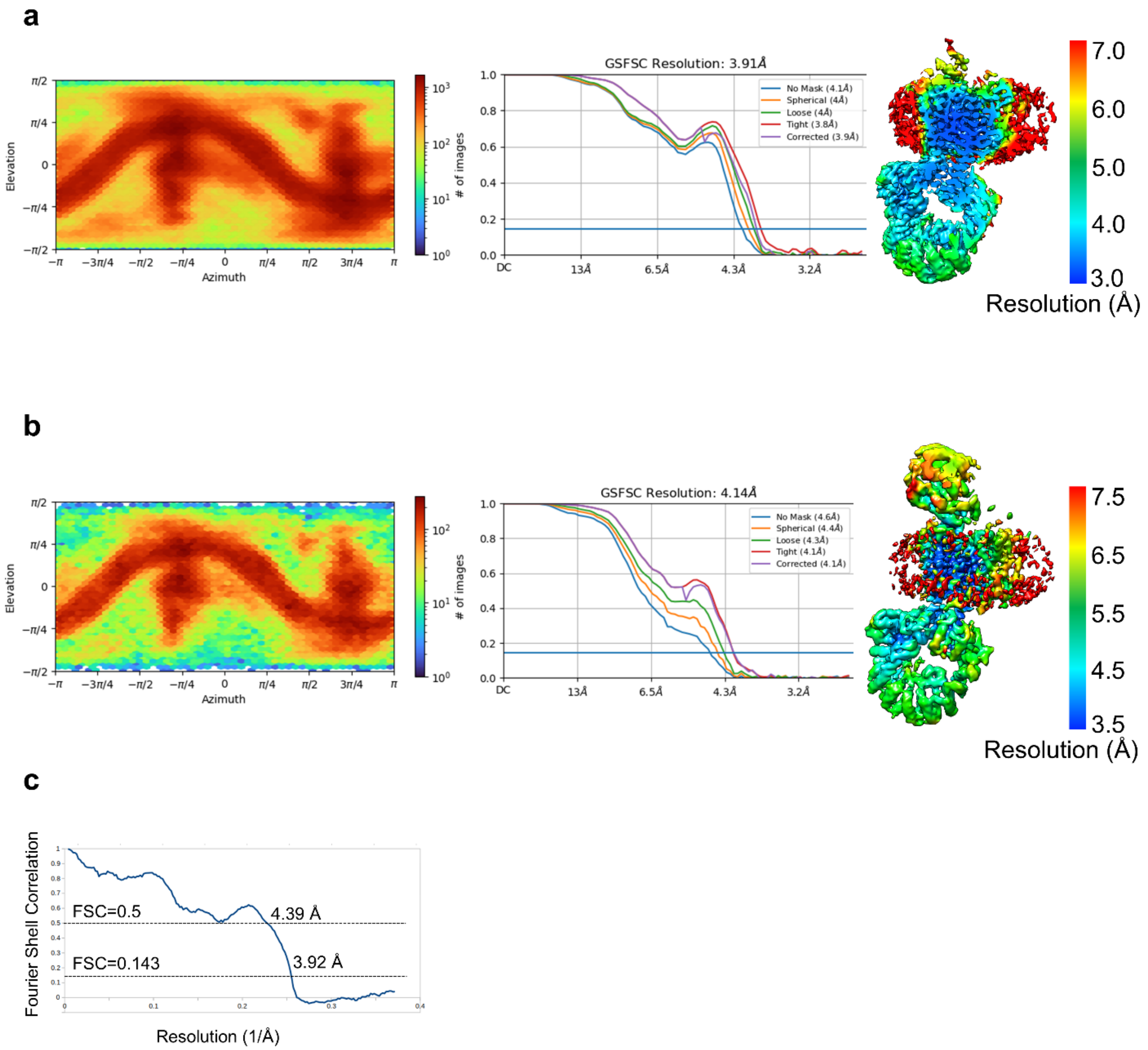

Supplementary Figure 6 | Angular distribution, Fourier shell correlation (FSC) curves, and local resolution of reconstruction maps. **a** Angular distribution, gold-standard FSC, and local resolution estimation of the 3.9 Å map. **b** Angle distribution, gold-standard FSC, and local resolution estimation of the 4.1 Å map. **c** FSC curve of map vs. model

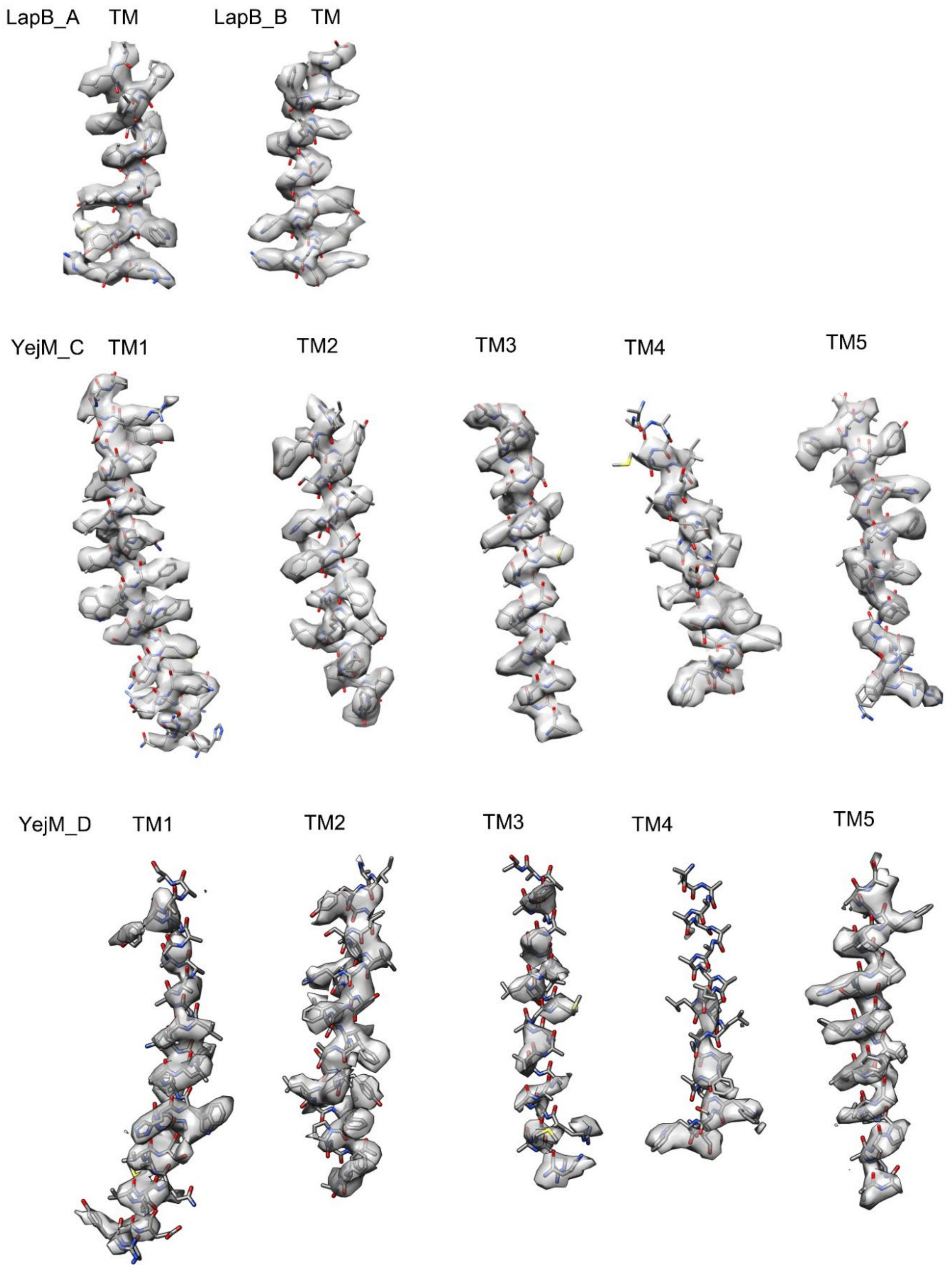

Supplementary Figure 7 | Densities and model of the TM domains in the YejM/LapB complex.

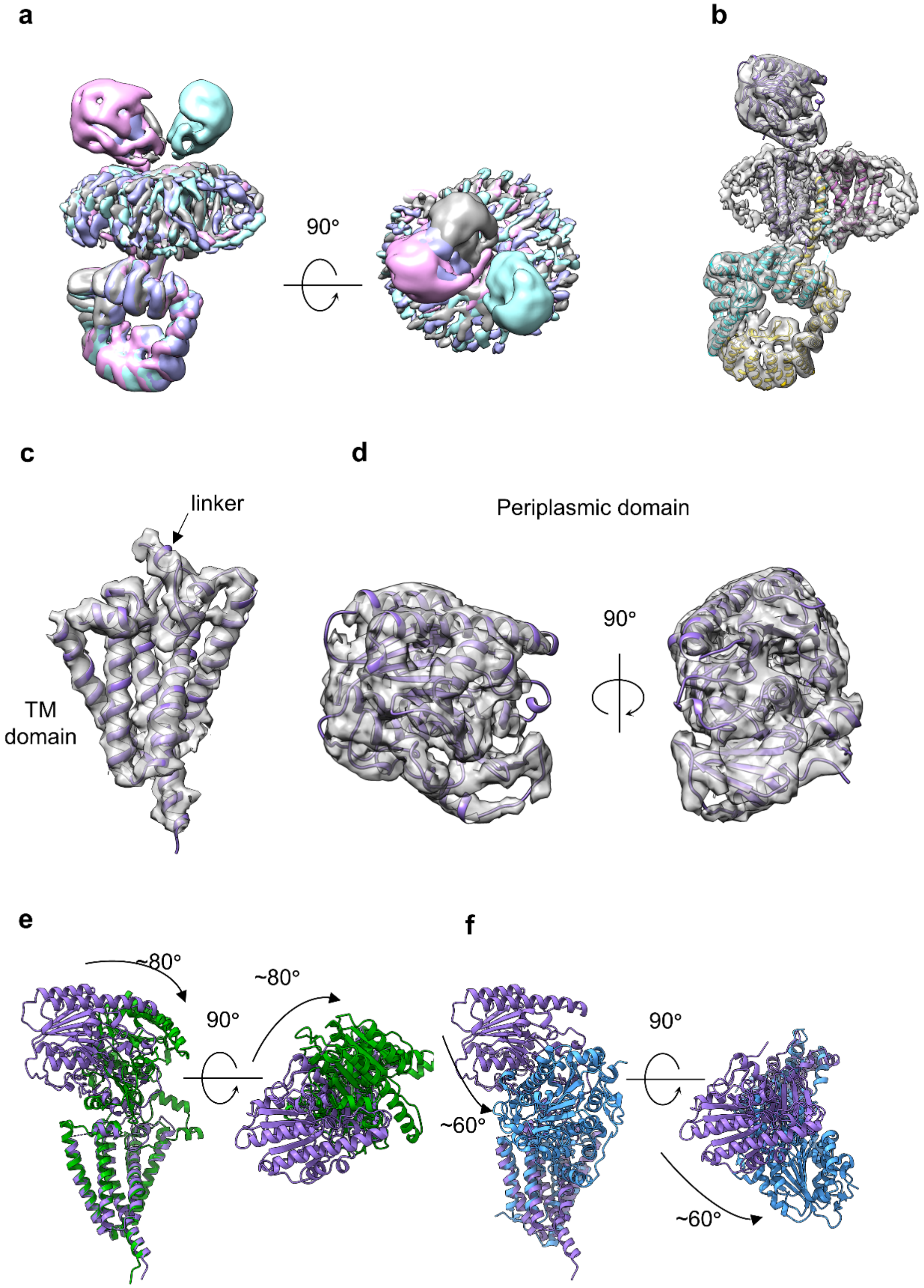

Supplementary Figure 8 | Flexibility of YejM periplasmic domain. **a** Superimposing maps of classes #1, #3, #4 and #5 from the second round of classification in Supplementary Fig. 4. **b** The

4.1 Å map with the fitted model. **c** The TM and linker regions of the 4.1 Å map with the fitted atomic model. **d** The periplasmic regions of the 4.1 Å map with a docked crystal structure of YejM periplasmic domain. **e** Structural comparison of YejM_C (purple) in the YejM/LapB complex with YejM in the YejM/LPS crystal structure (green) by superimposing their TM domains. **f** Structural comparison of YejM_C (purple) in the YejM/LapB complex with EptA (cyan) by superimposing their TM domains.

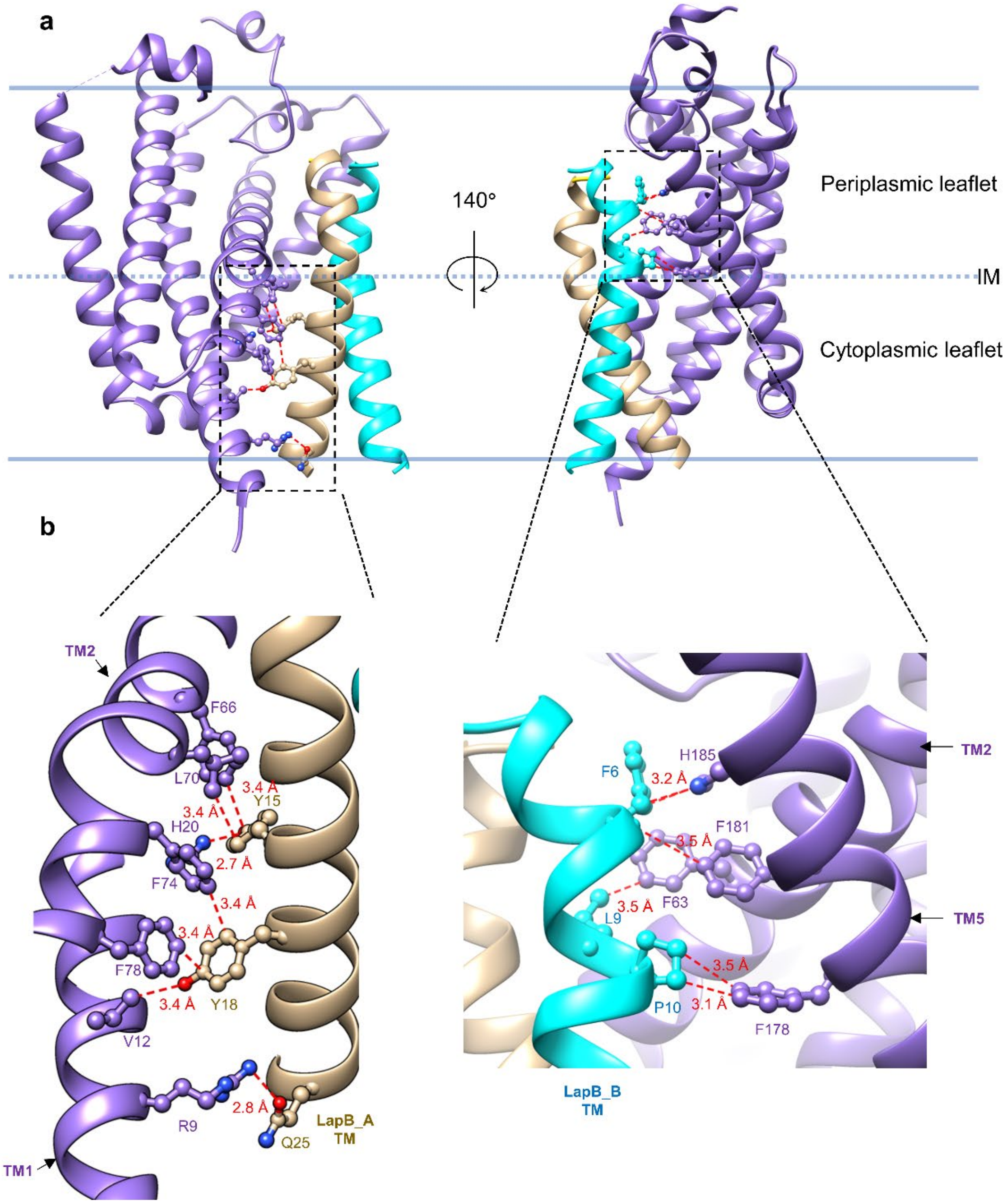

Supplementary Figure 9 | Interactions between YejM_C and LapB dimer. **a** Interactions between the YejM_C and each LapB molecule are located in the different leaflets of the IM. The thick blue lines indicate the boundaries of the inner membrane (IM) and the dashed line indicates the interface of two leaflets. Dashed rectangles highlight protein/protein interactions in each leaflet, with residues involved in interactions presented as sticks and balls. **b** Zoomed-in view of protein/protein interactions between YejM_C and the LapB dimer. Regions in the dashed rectangles are enlarged to show details of protein/protein interactions. Residues involved in interactions are labeled and shown as balls and sticks, and the interactions (within 3.5 Å) are highlighted by dashed red lines with distances labeled.

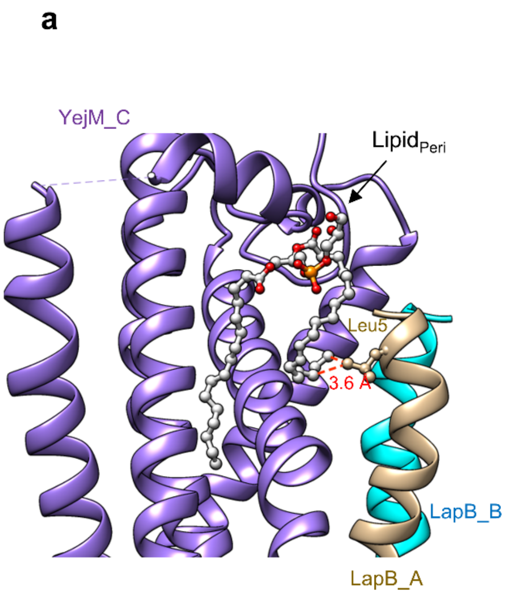

Supplementary Figure 10 | Interactions between the lipid molecules and LapB. Both lipids and the interacting residues are shown as ball and stick, and the interactions are highlighted by dashed red lines with distances labeled.

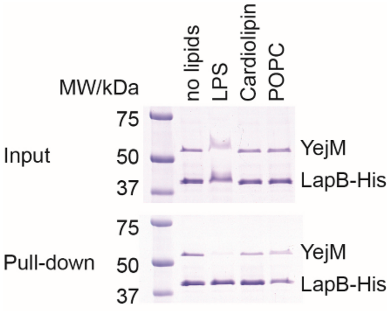

Supplementary Figure 11 | LPS disrupts the interactions between YejM and LapB. SDS-PAGE analysis of YejM/LapB$_{His}$, after incubating without or with lipids for 30 min (Input) and elution from TALON metal affinity resin after extensive washing (Pull-down). Each experiment was repeated independently three times with similar results. Source data are provided as a Source Data file.

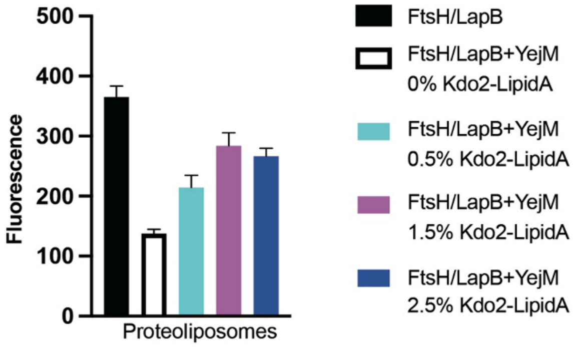

Supplementary Figure 12 | Dose-dependent effect of Kdo2-Lipid A on LpxC degradation by FtsH/LapB/YejM proteoliposomes. Each experiment was repeated three times and all data were presented as mean values with error bars representing standard deviations (SDs) of triplicates. Source data are provided as a Source Data file.

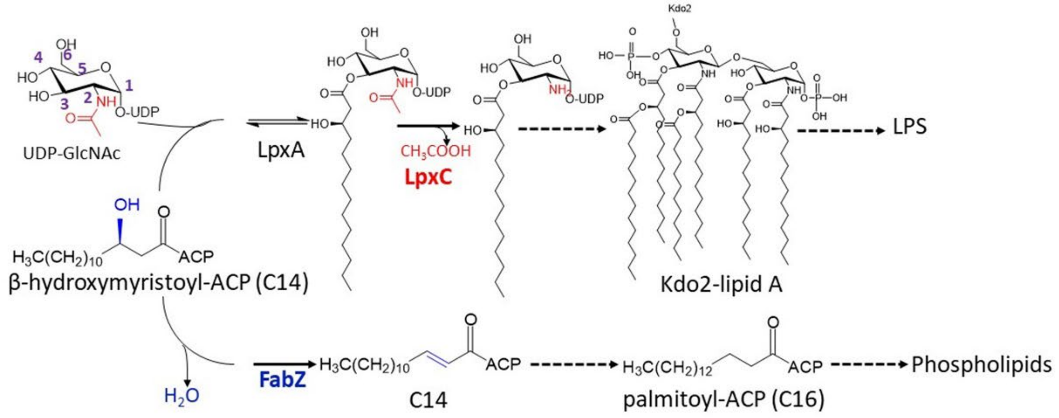

Supplementary Figure 13 | Coupling of LPS ad phospholipids synthesis. Two pathways share a common precursor, β-hydroxymyristoyl-ACP. Two key enzymes, LpxC and FabZ, are highlighted in two pathways. Multiple steps are skipped as dashed arrows for clarity.

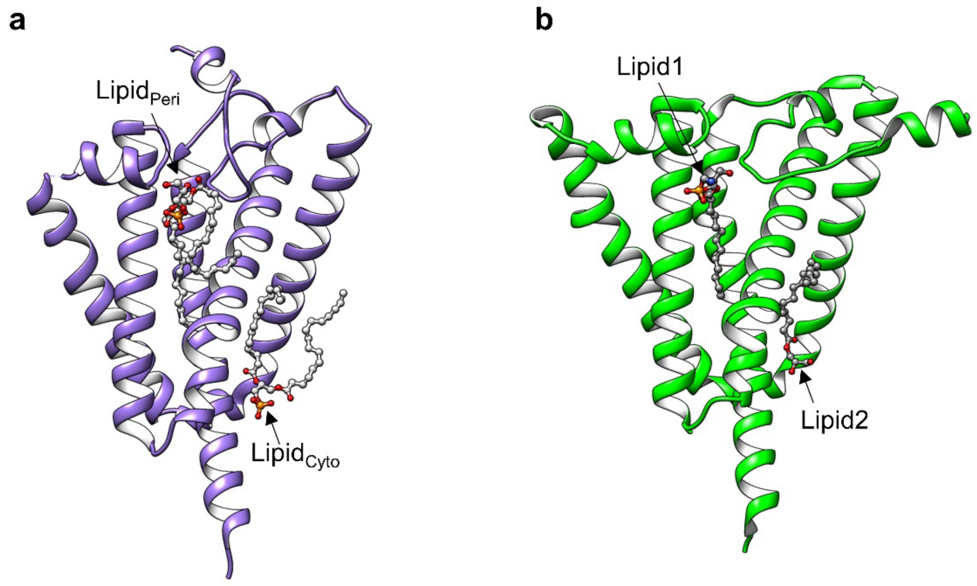

Supplementary Figure 14 | Phospholipid molecules in the YejM_C of the LapB/YejM complex and the crystal structure of YejM.

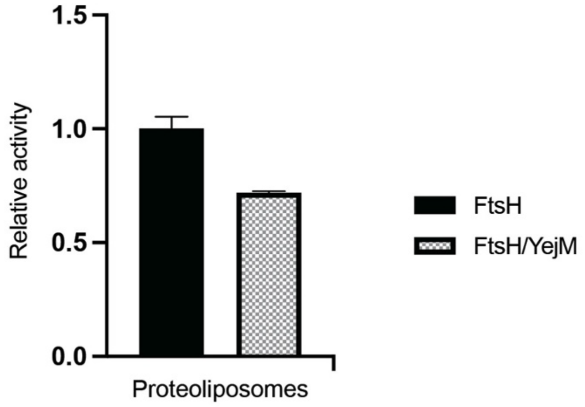

Supplementary Figure 15 | Protease activity on LpxC of proteoliposomes with FtsH and FtsH/YejM. The activity of FtsH-proteoliposomes is set to 1, and the activity of FtsH/YejM proteoliposomes is normalized to it. Each experiment was repeated three times and all data were presented as mean values with error bars representing standard deviations (SDs) of triplicates. Source data are provided as a Source Data file.

name	sequence	note
NdeI-FtsH-F	AGAAGGAGATATACAtatggcgaaaaacctaatactctgg	For pETduet-FtsH-3C-10His
FtsH-R	ACCCTGTGTTTGAGCCTCCTTGTCGCCTAACTGTTCTGAC	For pETduet-FtsH-3C-10His
FtsH-stop-F	CAGTTAGGCGACAAGtaaGAGGCTCAAACACAGGGT	Quick change to add stop code
FtsH-stop-R	CTGTGTTTGAGCCTCttaCTTGTCGCCTAACTGTTC	Quick change to add stop code
YciM-XhoI-R	GTGGTGGTGGTGGTGCtcCAGGCCATCAAGACCGC	For pRSF22b-LapB-6His
NdeI-YciM-F	AAGAAGGAGATATACATATGCTGGAGTTGTTGTTTCTG	For pRSF22b-LapB-6His
RSF-DjlA-F	AAGAAGGAGATATACATATGCAGTATTGGGGAAAAATCATTG	For pRSF22b-DjlA-TM-LapB-21-389-His
DjlA-TM-YciM21-R	TTGTTGCGCACTTCTGCGATGGCCAATTAACAGGCCTAAC	For pRSF22b-DjlA-TM-LapB-21-389-His
RSF-AcrZ-F	AAGGAGATATACATatgATGTTAGAGTTATTAAAAAGTCTGGTATTCG	For pRSF22b-AcrZ-TM-LapB-21-389-His
AcrZ-TM-YciM-R	TTGTTGCGCACTTCTGCGACCGTAAATCAGACCCAGG	For pRSF22b-AcrZ-TM-LapB-21-389-His
RSF-KdtA-F	AAGAAGGAGATATACATATGCTCGAATTGCTTTACACC	For pRSF22b-KdtA-TM-LapB-21-389-His
KdtA-TM-YciM21-R	TTGTTGCGCACTTCTGCGCACCCAGAGCCGTATCCAG	For pRSF22b-KdtA-TM-LapB-21-389-His
YciM-21-F	CGCAGAAGTGCGCAACAA	Amplify LapB 21-389
pET-LpxC-F	CACAGGGTCCTGGCGCcATGATCAAACAAAGGACACTTAAACG	For pETduet-N-10His-3C-LpxC
LpxC-AflII-R	ttactttctgttcgacTTATGCCAGTACAGCTGAAGGC	For pETduet-N-10His-3C-LpxC
pET-RpoHF	AAGAAGGAGATATACAtATGACTGACAAAATGCAAAGTTTAGC	For pETduet-RpoH-3C-10His
RpoH-3C-R	ACCCTGTGTTTGAGCCTCCTCGAGCGCTTCAATGGC	For pETduet-RpoH-3C-10His
pET-CII-F	CACAGGGTCCTGGCGCcATGGTTCGTGCAAACAAACG	For pETduet-N-10His-3C-λCII
pET-CII-R	TTACTTTCTGTTCGACTTAGAATTCCATCTGGATTTGTTCAG	For pETduet-N-10His-3C-λCII
NdeI-YciM21-F	AAGAAGGAGATATACATCGCAGAAGTGCGCAACA	For pRSF22B-LapBcyto-6His
NdeI-YejM-F	aagaaggagatatacatATGGTAACTCATCGTCAGCG	For pCDFduet-YejM-6His
YejM-His-XhoI-R	ggtttctttaccagactcagtgatggtggtgatggtgGTTAGCGATAAAACGCTTCTC	For pCDFduet-YejM-6His

Supplementary Table 1 | primers used in this study.

Protein or protein complex	Tag	Vector	Expression strain	Antibiotics	Medium	Expression temp./time	Detergent	Purification steps
FtsH	C-3C-10His	pETduet	BL21 Star^{TM} (DE3) pLysS	Amp Cam	TB	30°C, 4 hours	LMNG	Cobalt affinity purification
LapB$_{His}$	C-6HiS	pRSF22	BL21 Star^{TM} (DE3) pLysS	Kan Cam	TB	30°C, 4 hours	GDN	
FtsH$_{His}$/LapB$_{His}$	C-3C-10His	pETduet	BL21 Star^{TM} (DE3) pLysS	Amp Kan Cam	TB	30°C, 4 hours	LMNG	
	C-6His	pRSF22b						
FtsH/LapB$_{His}$	–	pETduet	BL21 Star^{TM} (DE3) pLysS	Amp Kan Cam	TB	30°C, 4 hours	LMNG	
	C-6His	pRSF22b						
FtsH/AcrZ$_{TM}$-LapB$_{cyto}$-His	–	pETduet	BL21 Star^{TM} (DE3) pLysS	Amp Kan Cam	TB	30°C, 4 hours	LMNG	
	C-6His	pRSF22b						
FtsH/DjlA$_{TM}$-LapB$_{cyto}$-His	–	pETduet	BL21 Star^{TM} (DE3) pLysS	Amp Kan Cam	TB	30°C, 4 hours	LMNG	
	C-6His	pRSF22b						
FtsH/KdtA$_{TM}$-LapB$_{cyto}$-His	–	pETduet	BL21 Star^{TM} (DE3) pLysS	Amp Kan Cam	TB	30°C, 4 hours	LMNG	
	C-6His	pRSF22b						
LpxC	N-10His-3C	pETduet	BL21 Star^{TM} (DE3) pLysS	Amp Cam	LB	30°C, 4 hours	–	Nickel affinity purification→3C cleavage→Flow throught Ni-NTA-agarose→SEC
RpoH	C-10His-3C	pETduet	BL21 Star^{TM} (DE3)	Amp	LB	18°C, overnight	–	
λCII$_{His}$	N-10His-3C	pETduet	BL21 Gold (DE3)	Amp	LB	37°C, 3 hours	–	Nickel affinity purification→SEC
LapB$_{cyto}$-His	C-6His	pRSF22b	BL21 Star^{TM} (DE3) pLysS	Kan Cam	LB	30°C, 4 hours	–	
YejM/LapB$_{His}$	–	pCDFduet	BL21 Star^{TM} (DE3) pLysS	Spec Kan Cam	TB	30°C, 4 hours	GDN	Cobalt affinity purification→SEC
	C-6His	pRSF22b						
YejM$_{His}$	C-6His	pCDFduet	BL21 Gold (DE3)	Spec	TB	18°C, overnight	DDM	Cobalt affinity purification

Supplementary Table 2 | constructs, expression conditions, and purification of the proteins/protein complexes

Cryo-EM data collection and processing	YejM/LapB without periplasmic domains	YejM/LapB with a periplasmic domain
Voltage (kV)	300	
Electron dose ($e^-/Å^2$)	50	
Number of collected movies	23,363	
Particle defocus range (µm)	1.0-2.5	
Number of particles for 3D classification	2,204,875	1,181,379
Number of particles for final map	1,181,379	175,387
Resolution (Å)	3.9	4.1
Map sharpening B-factor ($Å^2$)	-216	-167
**Atomic model refinement**		
Number of protein residues	1181	
Number of side chains	423	
Number of lipids	2	
Number of atoms	7566	
**Geometric parameters (RMSZ)**		
Bond length (Å)	0.32	
Bond angle (º)	0.50	
**Ramachandran statistics**		
Residues in favoured regions (%)	97.00	
Residues in allowed regions (%)	2.91	
Residues in disallowed regions (%)	0.09	
Rotamer outliers (%)	1.42	
MolProbity score	1.89	
Clash score	12.18	

Supplementary Table 3 | Statistics of the cryo-EM structures presented in this study.

REFERENCES:

54 Akiyama, Y. Self-processing of FtsH and its implication for the cleavage specificity of this protease. *Biochemistry* **38**, 11693-11699, doi:10.1021/bi991177c (1999).
