# Supplementary material for: Regulatory mechanisms of lipopolysaccharide synthesis in Escherichia coli
Source: Nat Commun. 2022 Aug 5;13:4576. doi: 10.1038/s41467-022-32277-1 (PMC9356133; doi:10.1038/s41467-022-32277-1)
Supplement: Supplementary file 3 — Description of Additional Supplementary Files [file 41467_2022_32277_MOESM3_ESM.pdf]

File name: Supplementary Movie 1

Description: **LPS and LapB dimer have overlapping binding sites in YejM.** Proteins are presented as cartoons (YejM in green, LapB in brown and cyan). LPS is presented as sticks
